# Home‐based high‐intensity interval training for people with Parkinson's: Protocol for the HIIT‐Home4Parkinson's randomized, controlled feasibility study

**DOI:** 10.1002/hsr2.1800

**Published:** 2024-01-07

**Authors:** Conrad Harpham, Hilary Gunn, Jonathan Marsden, Raul Bescos Garcia, Luke Connolly

**Affiliations:** ^1^ School of Health Professions University of Plymouth Devon UK

**Keywords:** exercise, HIIT, Parkinson's disease, rehabilitation, home‐based

## Abstract

**Background:**

High‐intensity interval training (HIIT) is feasible and beneficial for some people with Parkinson's (PwP), although adherence to extended programs may be problematic. PwP face barriers to exercise such as lack of time, expense, and difficulty with travel logistics due to motor symptoms. HIIT based in the home setting if practical, could therefore be apposite for PwP by overcoming these barriers. However, until now, no home‐based HIIT program for PwP has been developed. Cocreated by PwP, clinicians, and family members, the HIIT‐Home4Parkinson's (HH4P) program is an innovative, 12‐week home‐based HIIT program for PwP. This protocol describes a feasibility study designed to assess the feasibility and safety of the HH4P program, explore outcomes that may be sensitive to change, and inform the implementation of a potential full trial.

**Methods/design:**

Using a randomized controlled parallel group design, 24 independently mobile people with Parkinson's of mild to moderate disease severity will be randomized 1:1 to either the HH4P program plus usual care, or usual care alone. Both groups will be assessed at baseline, and upon the completion of the program. Outcomes will include feasibility and safety factors such as recruitment, completion, adverse events, and intervention fidelity with qualitative evaluation along with mechanistic, physiological, and clinical outcomes.

**Discussion:**

Results of this study will inform the rationale and methodological considerations for a full trial with long‐term follow‐up. Ultimately, further establishing the practicality and utility of home‐based HIIT could provide an important exercise option for PwP, potentially leading to extended participation and increased health and well‐being for this population.

## 1 BACKGROUND

Affecting 6.1 million people globally,^1^ Parkinson's disease (Parkinson's) is a neurodegenerative disorder characterized by motor impairments such as bradykinesia, postural instability, and rigidity along with a range of nonmotor symptoms.^2^ People with Parkinson's (PwP) can also demonstrate reduced aerobic fitness,^3^ potentially leading to additional health and well‐being complications. High‐intensity interval training (HIIT) is a common form of exercise, consisting of alternating bouts of high‐intensity exercise and periods of rest or active recovery. HIIT has been found to be feasible and safe for some PwP and can improve cardiorespiratory fitness, motor symptoms, and levels of brain‐derived neurotrophic factor (BDNF),^4^ evinced to have neuroprotective qualities.^5^ In addition, HIIT for PwP has been found to be at least as beneficial as moderate‐intensity continuous exercise, with reduced volume and time commitment,^4^ as reflected in other clinical and healthy populations.^6^, ^7^, ^8^ However, despite these benefits, PwP may not adhere to extended HIIT programs.^4^ PwP face restricting factors such as expense, concerns of exercising in a group setting, travel logistics due to motor symptoms,^9^ and lack of time.^10^ HIIT based in the home setting would appear to be a potential way to overcome these barriers, and possibly facilitate initial engagement and extended participation. However, until now, no home‐based HIIT program has been developed for PwP; therefore, the practicality and utility of home‐based HIIT remain unknown.

“HIIT‐Home4Parkinsons” (HH4P) is a novel, patient‐focused, cocreated home‐based HIIT program for PwP. HH4P will be delivered as an adaptable, individualized, 12‐week home‐based HIIT intervention, structured to maximize achievability through remote supervision and the use of appropriate physical and online resources. As previously described,^11^ the development of HH4P was based on the Medical Research Council (MRC) framework for the development and evaluation of complex interventions,^12^, ^13^, ^14^ continually informed by input from patient and public involvement (PPI) contributors and clinicians within a cocreative, iterative planning process of online focus groups and exercise testing. Having now optimally developed the HH4P program, it is critical to undertake a feasibility study before undertaking a definitive trial to address remaining uncertainties. This protocol describes the HH4P parallel‐group randomized controlled feasibility study, developed to evaluate the practicality and utility of the HH4P program, and ultimately inform the development of a full trial designed to facilitate the initial engagement and extended participation in HIIT for PwP.

This feasibility study has three aims.

1. To evaluate the feasibility, safety, and acceptability of a 12‐week home‐based HIIT program for PwP.
2. To identify the clinical and physiological outcomes that could be feasible and sensitive to change compared to usual care in a full home‐based HIIT trial for PwP.
3. To elucidate the key methodological considerations for the implementation of a full home‐based HIIT trial. Table 1 shows specific objectives relating to each aim.

**Table 1:** Feasibility study objectives.

Aim	Objectives. Aims will be achieved by determining
A	i. HH4P program safety ii. Adherence to the HH4P exercise program iii. Completion of the HH4P exercise program iv. Achieved exercise intensity v. Acceptance of HH4P exercise program and delivery procedures vi. Practicality of intervention resources vii. Intervention fidelity
B	i. Responsiveness to change in mechanistic, physiological, and clinical outcomes to inform the selection of primary and secondary outcomes for a definitive trial ii. Feasibility of outcome measure procedures (including rates of outcome measure completion) iii. The association between baseline and postintervention scores
C	i. Suitability and feasibility of eligibility criteria ii. Numbers of eligible participants from the target population iii. Willingness of patients to be randomized iv. Baseline factors most strongly associated with outcomes, to inform potential stratification in a full trial v. Recruitment/retention rates and suitability of procedures vi. Sample size calculation required for a fully powered randomized controlled trial vii. Resources required for a full trial

### 1.1 Registration

The HH4P randomized, controlled feasibility study was registered in clinicaltrials.gov, identification number NCT05485428.

## 2 METHODS/MATERIALS

This protocol was written in accordance with the SPIRIT 2013 checklist; recommended items to address in a clinical trial protocol and related documents,^15^ and the CONSORT 2010 statement: extension to randomized pilot and feasibility trials^16^ (see Supporting Information).

### 2.1 Study design overview

This is a randomized controlled parallel group feasibility study with mechanistic, physiological, and clinical subcomponents. Twenty‐four PwP will be randomized in a 1:1 ratio either to the HH4P 12‐week, home‐based HIIT program plus usual care (intervention) or to usual care alone (control). Assessments will include feasibility and safety factors, and pre and postintervention assessments of potential primary and secondary outcomes undertaken by assessors blinded to group allocation. Both groups will undergo objective physical activity (PA) monitoring 1 week before, and in Week 7 of the intervention. Exercise participants will be invited to attend a posttrial focus group constituting a qualitative addition to assessment of intervention fidelity.

### 2.2 Roles and responsibilities

- Research team:

The chief investigator (CI; author C.H.) has overall responsibility for the implementation of this trial. The CI has lead responsibility for writing ethics submissions, developing training materials, and recruitment, and will be supported by three members of the supervisory team (ST; authors L.C., H.G., and J.M.). The CI is also responsible for data analysis, participant liaison, online resources, and materials. One member of the ST (L.C.) will be allocated to undertake postrandomization outcome assessments, blinded to trial arm allocation. Another member of the ST (H.G.) who will not be involved in assessments will undertake randomization procedures.

- Trial steering committee

This study will be overseen by an independent trial steering committee (TSC). The TSC will consist of one service user (PwP), and a practicing clinician. The TSC will provide ongoing guidance and advice on any conflict that arises within the research team. The TSC will receive three progress reports delivered by the CI throughout the course of the study.

### 2.3 Setting

The HH4P program is based at the University of Plymouth (UoP), Devon, UK. All research assessments are currently scheduled to take place in the Exercise, Nutrition and Health Laboratory, Link Building, UoP. All HIIT sessions will take place in participant homes, expected to be located in the Southwest of England.

### 2.4 Participants

Participant eligibility criteria will include having Parkinson's with mild to moderate disease severity (Hoehn and Yahr Stages 1–3). This criterion was applied as a recent systematic review^4^ concluded there was insufficient evidence to suggest HIIT as a suitable exercise option for those of greater functional disability. An upper age limit will not be applied, as eligibility emphasis will be on appropriate functional and cognitive ability (Table 2).

**Table 2:** Participant eligibility criteria.

Inclusion criteria	Diagnosed with Parkinson's disease Aged 18 years or older (no upper limit) Hoehn and Yahr Stages 1–3 (mild to moderate disease severity) Capacity to consent under the Mental Capacity Act 2005, and sufficient cognitive ability to follow an exercise program Based at home with space to perform an exercise program (approximately 2 m^2^) Willing and able to travel to intervention assessments Access to a computer, Smart Phone, or tablet and to the internet
Exclusion criteria	Other concurrent neurological conditions Comorbidities that would prevent/be exacerbated by high‐intensity exercise such as forms of cardiovascular disease Advised to not participate following medical consultation Participation in a contemporaneous interventional study

### 2.5 Sample size

As this is a feasibility study, an a priori power calculation to detect a significant between‐group difference in a primary outcome is not appropriate.^17^ Instead, this study aims to gain estimates of the likely rates of recruitment and retention, and variability of the proposed primary and secondary outcomes to inform sample size calculations for a potential full trial (objective Cvi). For this feasibility study, we aim to recruit 24 participants^18^ (12 interventions and 12 usual care controls). It is anticipated that to recruit these 24 participants we will need to telephone screen approximately 37 people, of which approximately 35% (*n* = 13) will be found to be ineligible after screening, leaving 24 people to grant consent, and ultimately be randomized. From other similar studies,^19^ we believe that retention rates will be at least 85%, leaving a likely sample at the final follow‐up of between 20 and 24 participants.

### 2.6 Recruitment strategy

This feasibility study will aim to recruit from the Southwest of England over a 3‐month period, but participants from other regions are eligible if they are willing/able to travel to the UoP for assessments.

Participants will be identified through the following sources:

1. University Hospitals Plymouth National Health Service Trust Parkinson's Service (clinicians will pass on promotional materials during scheduled appointments)
2. Livewell Southwest (as above)
3. Parkinson's UK (promotional material will be emailed to members)
4. University of Plymouth social media outlets

All promotional materials will include information inviting people to email expressions of interest to the CI. Upon receipt of interest, potential participants will be directed to a Jisc online participant information sheet (PIS), including estimated start and completion dates and a short questionnaire. Participants will complete personal and telephone contact details and a statement of permission to undertake telephone screening.

### 2.7 Screening process

The CI will telephone potential participants to answer any further questions and to screen for eligibility using a preformatted screening checklist based on the eligibility criteria. Once eligibility is established, an appointment letter for baseline assessments will be sent via email by the CI. This will include information that assessments will be undertaken in the “on” medication state, and to time medication accordingly. During the baseline assessment, eligibility will be confirmed in person, with signed consent, assessment of blood pressure (BP), and a bespoke health screening questionnaire completed.

### 2.8 Recruitment rate

This feasibility study will help to elucidate the recruitment rate for the planned definitive trial as we will record the sources of recruitment for all participants in this study, and ensure that the distribution is considered when extrapolating likely recruitment rates for the main trial.

**Figure 1:**
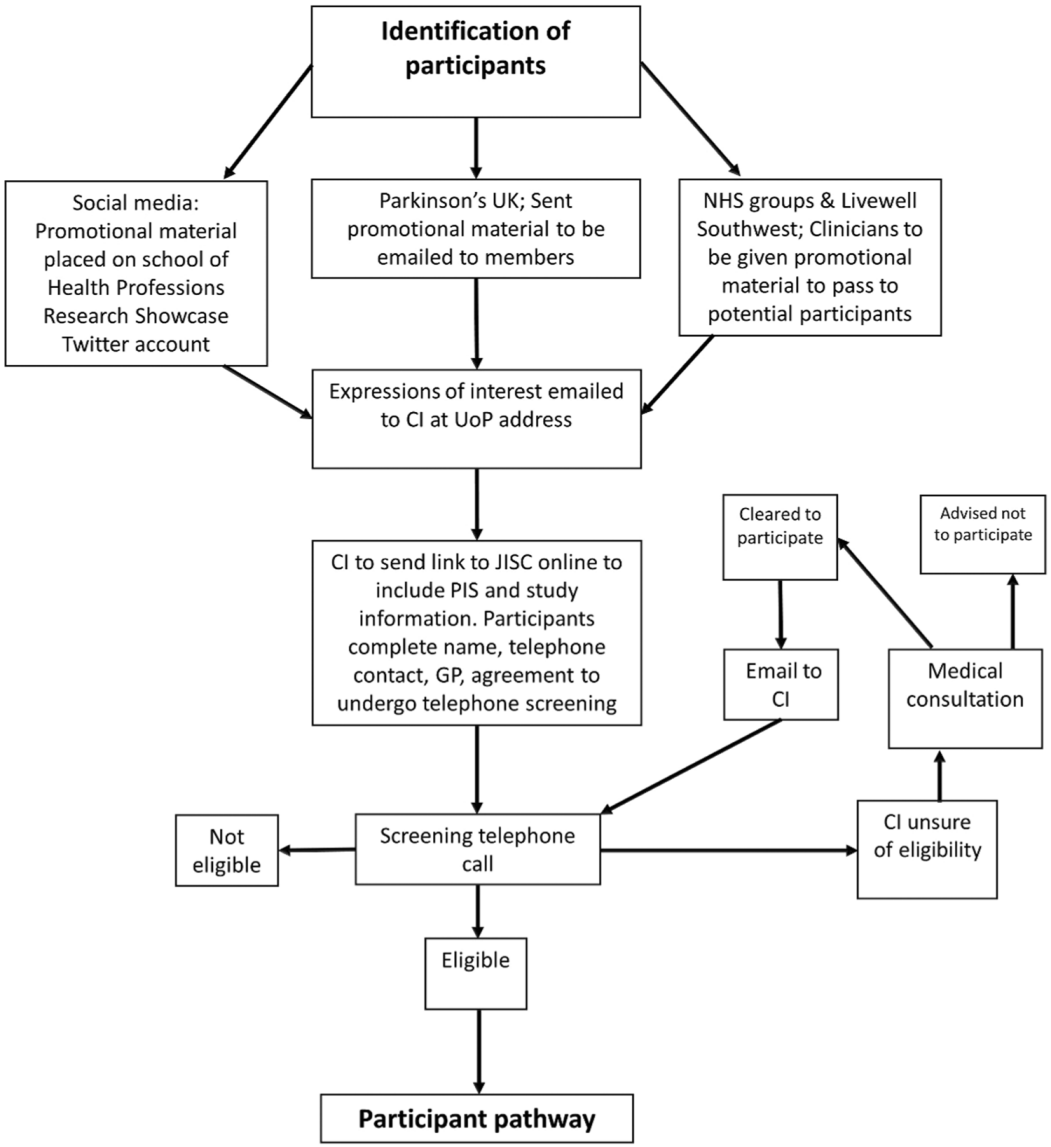
Recruitment pathway. CI, chief investigator; GP, general practitioner; NHS, National Health Service; PIS, participant information sheet; UoP, University of Plymouth.

The recruitment period for this feasibility study is 3 months. The HH4P recruitment pathway can be seen in Figure 1.

### 2.9 Consent

There will be three layers of consent to this study:

1. Permission to be screened:

PwP interested in participating will be invited to complete an online form which includes a statement of permission to undertake the telephone screening process.

2. Consent to be recruited to the feasibility study:

Written informed consent will be obtained by the CI at the baseline assessment session before any data collection. Having been previously provided with study information via Jisc online, participants will have had the opportunity to review the information sheet and ask any questions before attending the baseline assessment session. Consent will also be sought to contact patients' GP to inform them of participation in the trial.

3. Consent to participate in posttrial focus groups:

Participants will be informed of the postexercise program focus group within the PIS, and consent will be included within the prebaseline informed consent process.

### 2.10 Assessment sessions and home visits

All participants will attend two assessment visits to the UoP. The first assessment visit will take place 1 week before the start of the intervention to collect baseline data and ascertain preintervention outcome scores. Participants will then be given preprogrammed PA monitors, to objectively measure PA for a period of 7 days. During this week, a blinded member of the ST (H.G.) will randomly allocate the participant to either the HIIT or control group.

After this 7‐day period, the CI will undertake a home visit to collect PA monitors, provide HIIT participants with resources, inform participants of group allocation, and provide both groups with instruction (see Harpham et al.^11^ for details). All participants will be reminded that allocation to either intervention or control arm of the trial was informed by chance and occurred after baseline assessment.

**Figure 2:**
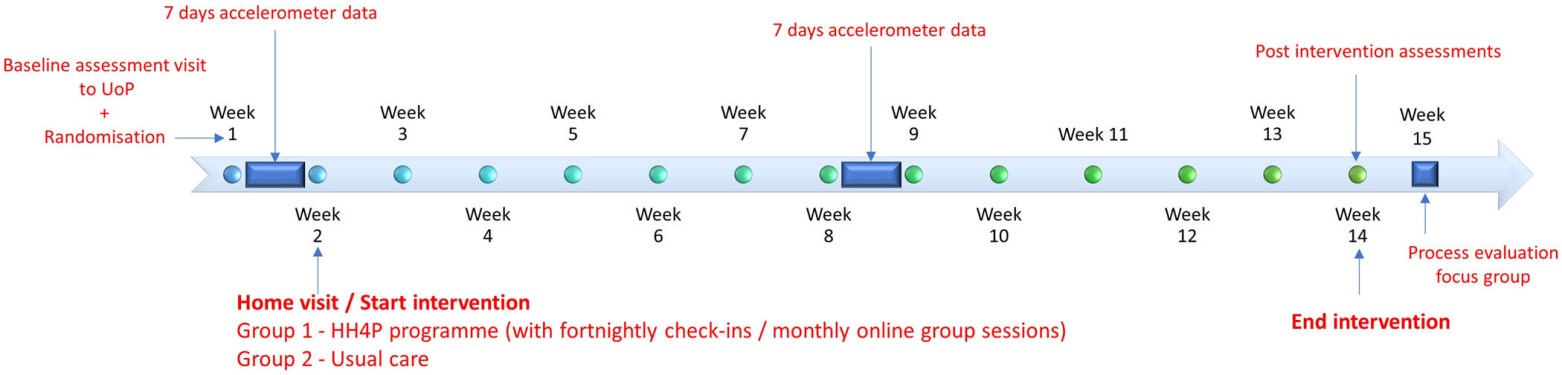
Timeline of study events and assessments. HH4P, HIIT‐Home4Parkinson's; UoP, University of Plymouth.

The second assessment visit to ascertain postintervention outcome scores will be 24 h after the completion of the program. Figure 2 shows a timeline of study events and assessments.

### 2.11 Outcomes

#### 2.11.1 Baseline data

Baseline data will be collected at the initial assessment visit during which eligibility is confirmed and once informed consent is obtained/bespoke health screening questionnaire is completed. The initial assessment visit will take place 1 week before the start of the HH4P program delivery date. The following baseline data will be collected:

- Demographic data: Gender, age, ethnicity, home circumstances (who they live with), and employment status (as indications of socioeconomic status).
- Anthropometric data: Body mass (kg), height (cm), and body mass index will be collected/calculated with validated protocols, using a calibrated stadiometer and digital scales.
- Lifestyle data: Smoking status.
- Diagnostic data: Hoehn and Yahr stage.^20^
- Medications and comorbidities: All current prescribed medications and dosage will be listed and coded.
- Blood pressure: If BP results are abnormal according to clinical guidelines,^21^ the participant will rest for 10 min. BP will then be remeasured. If BP is still abnormal the participant will be asked to seek medical appraisal before reapplying for the study. Also, participants will be constantly visually monitored during assessment tests for changes in BP. Guidance on symptoms related to extreme rises or drops in BP will be included in the home exercise support documentation.

### 2.12 Feasibility outcomes

Measures of adherence and engagement:

- Program completion (Objective Aiii)

Program completion will be defined as the percentage of participants that completed the 12‐week program of exercise or usual care, from those that underwent initial randomization.

- Attendance at online group sessions (Objective Aii)
- Frequency and duration of exercise sessions (Objective Aii)
- Web‐based participant heart rate (HR) monitoring (Objective Aii)

Engagement in the home‐based program (exercise choice, HIIT session frequency, and duration) will be monitored based on exercise diary data recorded by the participant over the 12‐week program. Adherence will also be monitored through continual observation of online HR data (see below) and through attendance at online group meetings.

### 2.13 Process effectiveness

- Achieved HR (Objective Aiv)

Establishing the ability of participants to achieve the appropriate HR will be a key aspect within the evaluation of home‐based HIIT feasibility. Exercise participants will be supplied with a Polar H9 HR monitor, to be used during every home‐based HIIT session. HR will be recorded using the Polar Beat Smart Phone application (downloaded on participant Smartphones), paired to a Bluetooth‐equipped Polar H9 HR monitor, with each session uploaded to Polar Flow online software, and exported as a Microsoft Excel spreadsheet (version 2204). Achieved HR will also be recorded in participant diaries at the end of each HIIT set (three bouts of 45 s).

- Rate of perceived exertion (Objective Aiv)

As other factors can affect exercise intensity in PwP, such as motor symptoms and/or deconditioned muscle affecting movement economy,^22^ the self‐reported rate of perceived exertion (RPE) will also be recorded with the 6–20 point Borg Scale^23^ as a secondary measure of achieved intensity. Participants will record RPE in diaries at the end of each HIIT set.

### 2.14 Safety

- Adverse effects and events (Objective Ai; also refer to Section 2.23)

Adverse effects and events will be the primary measure of safety, which will contribute to the assessment of the practicality of HIIT in the remotely supervised home setting. Adverse effects and events will be patient recorded in participant diaries throughout the 12‐week program, and reported to the CI during fortnightly online drop‐ins.

### 2.15 Proposed primary outcomes for a full trial

- Mechanistic biomarker: Change from baseline blood serum BDNF (Objectives Bi–Biii, Civ)

Recent evidence suggests that high‐intensity exercise, including HIIT can increase circulating levels of BDNF,^24^ a member of the neurotrophin family of growth factors evinced to have several neuroprotective qualities.^5^ Preintervention blood collection will be undertaken before VO_2max_ testing and other clinical tests, as acute high‐intensity exercise can result in a transient increase in serum levels of BDNF that can take up to 2 h to return to baseline.^25^ Similarly, postintervention measurement will be undertaken at least 24 h after the final HIIT session. Blood serum will be analyzed in favor of blood plasma, as plasma BDNF has been suggested as a more reliable measure for clinical studies due to fewer potential technical influences during blood sample processing.^26^

Blood collection, transportation, and analysis: Two blood samples (6 mL) will be taken by a fully trained phlebotomist during each assessment visit. Samples will be allowed to clot at room temperature for 30 min,^27^ then rapidly centrifuged at 4000 revolutions per minute for 10 min at 4°C. Serum will be pipetted into microtubes (1.5 mL), labeled with the participant study code, date, time, and pre‐ or postintervention. Tubes containing serum will be immediately stored at −80°C, pending analysis.

Sample transportation and BDNF analysis: Analysis will start upon the completion of the HH4P program and collection of all data. Samples will be analyzed at Manchester Metropolitan University (MMU), by the CI, one member of the ST (L.C.) and one researcher from MMU. A transfer agreement will be written and signed by the UoP and MMU before shipping microtubes with a professional courier and following the guidance for dry ice shipments. Samples will be analyzed with the BDNF sandwich enzyme‐linked immunosorbent assay kit (Promega).

- Progression of motor symptoms: Change from baseline Movement Disorder Society Unified Parkinson's Disease Rating Scale part III (Objectives Bi–Biii, Civ).

The Movement Disorder Society Unified Parkinson's Disease Rating Scale (MDS‐UPDRS) is a clinician and patient‐completed rating tool designed to assess disease severity and progression. The MDS‐UPDRS part III focuses specifically on motor aspects such as rigidity and gait, is evidenced to be internally valid,^28^ and will be utilized in accordance with the official UPDRS III protocol.

- Cardiorespiratory fitness: Change from baseline maximal oxygen uptake (VO_2max_) (Objectives Bi–Biii, Civ)

Increased cardiorespiratory fitness is associated with a myriad of putative health benefits in both healthy and clinical populations. However, cardiorespiratory fitness is commonly reduced in PwP,^3^ potentially leading to further health and well‐being complications.

VO_2max_ will be measured by undertaking an incremental exercise test (IET) using a Lode Corival cardiopulmonary exercise testing cycle ergometer. Oxygen consumption and carbon dioxide production will be measured with a face mask connected to a calibrated Cortex 3B‐R3 (Cortex Biophysik). The IET protocol will be as previously described.^11^ Due to the maximal nature of the IET and the potential to influence other results,^25^ VO_2max_ testing will be undertaken last.

### 2.16 Proposed secondary outcomes for a full trial

- Lower extremity strength; change from baseline 30 s sit to stand test (30 s STS) (Objectives Bi‐Biii, Civ)

The 30 s STS, commonly used for assessing lower body strength and balance control in older adults and PwP, measures the number of times a participant can stand from a seated position in 30 s. The 30 s STS represents an important functional outcome, as reduced lower limb strength has been associated with both lower exercise and walking capacity in PwP.^29^, ^30^

- Activities of daily living: Change from baseline Oxford Participation and Activities Questionnaire (OxPAQ) Acute (Objectives Bi–Biii, Civ)

The OxPAQ acute is a patient‐centric, 23‐item self‐reported questionnaire consisting of items pertaining to activities of daily life and participation, used for patients with a variety of health conditions including Parkinson's. The OxPAQ acute varies from the original OxPAQ in that items refer to a reduced recall period of 1 week compared to four in the original, considered appropriate for this feasibility study to extract a more accurate response from participants. A license to use the OxPAQ acute was kindly granted by the Oxford University Innovation Clinical Outcomes Service.

- Maximum HR (HRmax) (Objective Aiv)

HR_max_ will be measured pre‐intervention only, during VO_2max_ testing with a Bluetooth‐equipped Polar H9 HR monitor (Polar). This will enable the calculation of individualized HIIT minimum target intensity (75% HR_max_).^11^

- Physical activity level (Objectives Bi and Biii)

As an important confounding variable, participant PA will be objectively measured for both trial arms with activePAL^TM^ accelerometers (activPAL™; Paltechnologies Ltd) for a 7‐day period before the program following initial baseline assessments, and also during week 7 of the program.

### 2.17 Process evaluation (Objectives Avii and Cvii)

The following elements of process evaluation are included in this feasibility study, guided by the MRC Process Evaluation of Complex Interventions Guidelines.^13^

Fidelity testing: Data from participants' diaries (exercises undertaken, session frequency and duration, RPE) and HR data will be used to evaluate the quality of the intervention delivery and the degree of concordance between the HH4P protocol and the actual program delivery.

### 2.18 Qualitative evaluation: participant focus group (Objectives Av, Avi, and Ciii)

Fidelity testing will be supplemented with the inclusion of a qualitative aspect,^13^ to explore participant perceptions of the intervention. A semistructured focus group (or groups, depending on numbers) will be undertaken involving all exercise participants after final outcome measures.

Focus groups will explore the acceptance of HH4P exercise program and delivery procedures, practicality of intervention resources, and willingness of participants to be randomized. Any possible adaptations will also be identified.

### 2.19 Other assessments

- Identification of how and why participants are “lost to follow‐up.”
- Measures taken to obtain the information if visits or data collection time points are missed.

If an assessment visit is missed, the CI will book another appointment, preferably within 1 week of the original date.

- Outcome data that will be recorded from protocol nonadherers.

As far as possible, all outcome data will be collected for all participants, regardless of whether or not they have adhered to the protocol to enable intention‐to‐treat (ITT) analysis.

### 2.20 Randomization

Initially, participants will be numbered in the order in which they give written consent. It is expected that recruitment will proceed gradually throughout the 3‐month window, therefore randomization by minimization will be undertaken during the recruitment process to either the HH4P or the usual care control group. The inclusion of a control group will allow home‐based HIIT utility evaluation by exploring changes in outcomes with the most robust, internally valid methodology available. The randomization procedure will be programmed before any participant recruitment into “Minim” MS‐DOS minimization software, to include a 1:1 ratio and group stratification by disease severity and gender. Upon recruitment of each participant and following their baseline assessments, randomized group allocation will be undertaken by a third member of the ST (H.G.), blinded to assessment results. Following this, an email will be sent by H.G. to the CI to notify them of each participant's allocated group; the CI will then notify the participant at the initial home visit.

### 2.21 Blinding

Participants are unable to be blinded due to the nature of the exercise intervention. Similarly, the CI is unable to be blinded. However, initial baseline assessments for each participant will be undertaken before randomization, and the member of the ST (L.C.) undertaking postintervention outcome assessments will be blinded to the participants' allocated group. All assessments will be undertaken in visits arranged independently of any intervention sessions, and away from the participant's home. Every effort will be made throughout to ensure these assessments are blinded, for example, by reminding participants not to discuss their group allocation with the blinded member of the ST. The final unblinding of the ST member who undertook assessments will be after the creation of a locked data set and analysis has been undertaken.

### 2.22 Interventions

- HH4P (HIIT + usual care).

The HH4P program is a 12‐week, thrice weekly HIIT program with the emphasis on providing options to facilitate individual preference, ability, and disease stage. As previously described in detail,^11^ HIIT sessions will comprise four sets of three 45 s bouts of high‐intensity exercise (≥75% HR_max_), with each bout interspersed with 15 s of rest. A 2‐min rest period will follow each set. HIIT sessions will last for 32 min each including warm‐up and cool‐down periods. Participants will choose from a range of exercises and will be supported by both physical and online resources.

- Usual care control group

All participants allocated to this group will continue to receive their usual clinical care; thus, with the exception of the trial assessments they will not be asked to attend any additional visits or sessions.

Both the HIIT and control group will be asked to not begin additional structured physiotherapy or exercise sessions, or undertake contemporaneous interventional studies.

### 2.23 Management of adverse and serious adverse events

The adverse event (AE) risks of taking part in this trial have been assessed to be low. However, all participants will be asked to report any new or worsening problems that they perceive to be related to participation in activity and/or exercise, as well as any relapses in their diaries. During fortnightly check‐ins, the CI will overtly request any information regarding AE's. These will then be recorded on the AE report form and reported by the CI to the ST. AE's considered related to the trial intervention will be followed until resolution or the event is considered stable. It will be left to the judgment of the CI, ST, and TSC, whether or not an AE is of sufficient severity to necessitate the participant to withdraw from the HH4P intervention.

Any serious adverse event (SAE), whether thought to be related to any trial intervention or not, will be reported by the CI to the ST within 24 h of the CI becoming aware of it. If the CI and ST consider that the SAE is not, or is unlikely to be, related to the trial, they will obtain a second assessment of causality from the TSC. SAE's which in the opinion of either adjudicator are possibly related to the trial intervention and unexpected will be reported by the CI to the Faculty of Health Research Ethics and Integrity Committee (FoHREIC) and Health Research Authority/Research Ethics Committee (HRA REC) within 15 days of having become aware of the event.

### 2.24 Withdrawal from the study

Any participant may at any time after they have consented decide that they wish to withdraw from continuation in the study (or following advice from a medical professional), which may also include withdrawal of all data and/or blood samples. In line with CONSORT Guidelines^31^ reasons for withdrawal will be recorded.

### 2.25 End of trial

The end of trial is the date of the completion of the postintervention focus group. The following criteria will be used to prematurely stop the research:

1. A decision made by the CI or any member of the ST on the grounds of safety issues, such as an unacceptable number of adverse events.
2. An evaluation via a fully powered RCT of a similar home‐based HIIT program for PwP. There are currently (last assessed June 21, 2023) no similar trials registered with clinicaltrials.gov.

**Figure 3:**
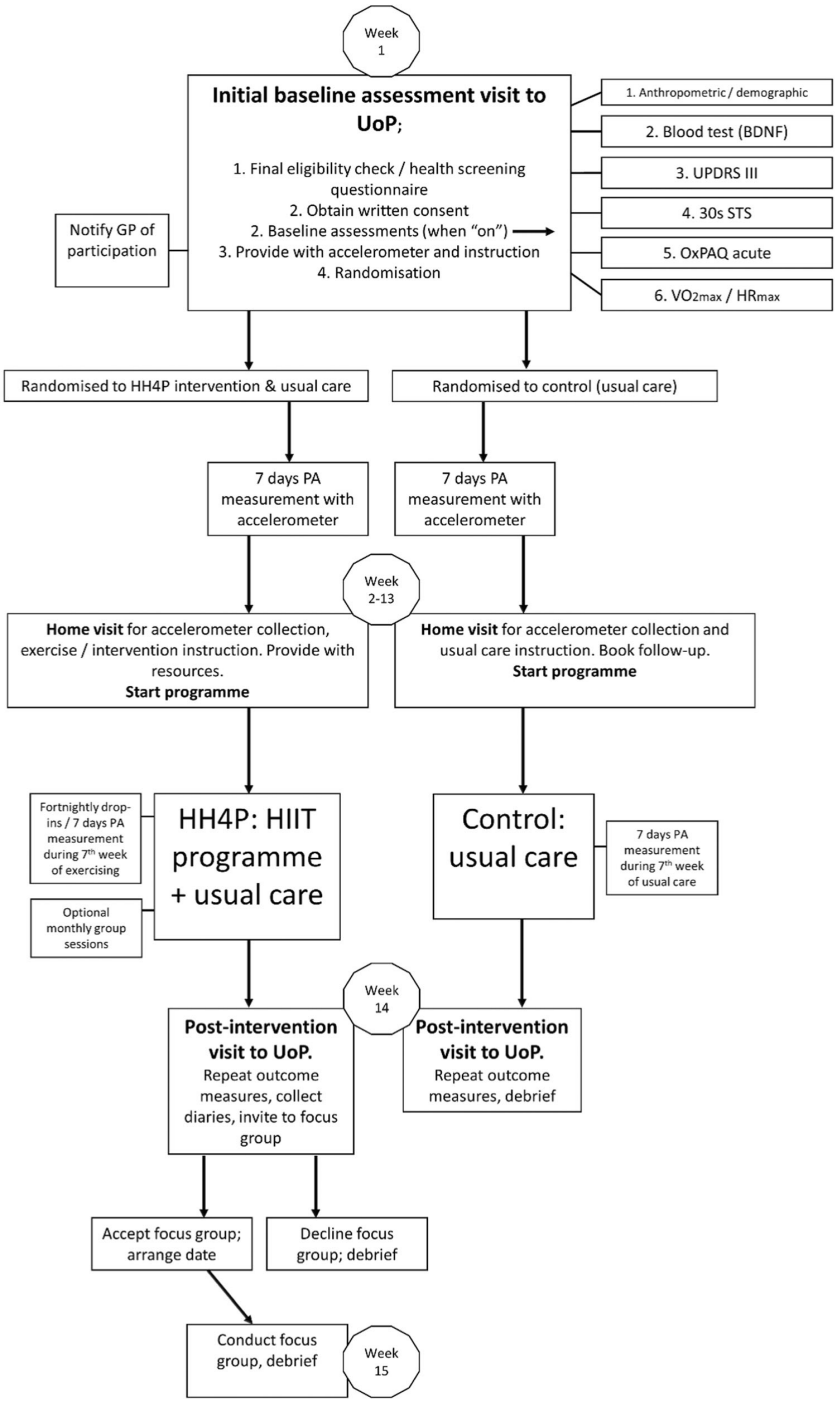
Overview of anticipated participant pathways. BDNF, brain‐derived neurotrophic factor; GP, general practitioner; HH4P, HIIT‐Home4Parkinson's; HIIT, high‐intensity interval training; HR_max_, maximum heart rate; OxPAQ, Oxford participation and activities questionnaire; PA, physical activity; STS, sit to stand; UoP, University of Plymouth; UPDRS, Unified Parkinson's Disease rating scale; VO_2max_, maximal oxygen uptake.

Figure 3 shows a schematic overview of the HH4P feasibility study participant pathways.

### 2.26 Data analysis

#### 2.26.1 Quantitative analysis

A detailed analysis plan will be developed before any statistical analysis; however, the below provides an overview of the expected plan;

- Summary of baseline data and flow of participants.

A CONSORT diagram will provide detailed descriptions of numbers of expressions of interest, meeting eligibility, having baseline data collected, being randomized, receiving the intervention, and having post‐intervention data collected.

- Outcome analysis.
  Analyses of quantitative data will be in two stages:
  1. Feasibility outcomes: Data from screening and recruitment will be used to generate estimates of eligibility, recruitment, retention, and consent in the trial population (Objectives ciii and cvii). Adherence data will also be used to assess the acceptability of the HH4P program (Objective Av). Estimation of pre‐ and postintervention completion rates for mechanistic, physiological, and clinical outcome measures will be undertaken along with appropriate confidence intervals (Objective Bii). In addition, Spearman's rank correlation coefficient will be calculated to assess the robustness of each outcome, by comparing pre and postintervention scores (Objective Bii). Furthermore, multiple regression will be used to explore the baseline factors that are most strongly associated with outcomes, to inform potential stratification in a full trial (Objective Civ).
  2. Mechanistic, physiological, and clinical outcomes: As it is inappropriate to use feasibility study data to formally test for between‐group treatment effects, the mechanistic, physiological, and clinical outcome analyses will primarily be of a descriptive nature, rather than inferential.^17^ Descriptive statistics of the proposed primary and secondary outcomes will be produced, as appropriate for each measure for each trial arm. Interval estimates of the potential intervention effects, relative to usual care only, will be produced in the form of a 95% confidence interval (Objective Bi). Missing data will be imputed using a measure of central tendency to allow ITT analysis.

IBM SPSS software (version 27) and Microsoft Excel (version 2209) will be utilized for quantitative data analysis.

### 2.27 Qualitative analysis

Postintervention focus groups will be undertaken with Zoom online (Zoom Video Communications), recorded on the UoP secure OneDrive, and transcribed verbatim by the CI. Transcripts will be imported into NVIVO software (QSR International) and analyzed with the use of thematic analysis.^32^, ^33^ Through a deductive semantic approach, a coding framework will be developed to recognize emerging themes relating to predefined topics for discussion. Data will be arranged into major thematic categories and subcategories, with the findings presenting specific factors relating to the acceptability of HH4P delivery and procedures (Objective Av), practicality of intervention resources (Objective Cii), and the willingness of patients to be randomized (Objective Cv).

### 2.28 Ethics

This study will be conducted in accordance with the Declaration of Helsinki, 1996.^34^ The study will not be initiated before the protocol, informed consent forms, participant information sheets, and other relevant documents (e.g., advertisements) have received ethical approval from the FoHREIC and HRA REC.

### 2.29 Protocol amendments

Should a protocol amendment be made that requires ethical approval, the changes in the protocol will not be instituted until the amendment and revised informed consent forms and PIS (if appropriate) have been reviewed and received the necessary approval. A protocol amendment intended to eliminate an apparent immediate hazard to participants may be implemented immediately providing that the HRA REC is notified as soon as possible and an approval is requested.

Minor protocol amendments only for logistical or administrative changes may be implemented immediately, and the HRA REC will be informed.

### 2.30 Data protection and confidentiality

All researchers will comply with the requirements of the Data Protection Act 2018 with regard to the collection, storage, processing, and disclosure of personal information. Electronic trial records will be stored on the secure UoP OneDrive, accessed only with a pass‐protected UoP laptop. For further information, please refer to the HH4P data management plan, publicly available at https://dmponline.dcc.ac.uk/.

### 2.31 Poststudy care

All individuals involved in the feasibility study will continue to receive the usual care that they would receive once the study has ceased. Participants in both the intervention and control arm of the study will have ongoing access to web‐based resources after the study.

### 2.32 Progression to a full trial

A full trial application will be made if minimum success criteria are achieved in the key aims and objectives. These criteria will be finalized in discussions involving the CI, ST, and TSC and could include:

- a minimum of 80% of consented participants randomized to the intervention group engaging with the 12‐week HH4P intervention.
- a minimum of 80% completion rate of key outcome measures (pre and post).

There are also other areas such as participant acceptability, and feasibility of study procedures that will be taken into consideration when deciding whether to progress to a full trial application.

### 2.33 Dissemination

This study is intended to inform the implementation of a full trial, rather than clinical decision‐making. Therefore dissemination will involve publication of the feasibility study results following relevant guidelines,^16^, ^35^ focusing on methodological considerations previously outlined in the study aims and objectives. Also, participants and contributing organizations will be offered a summary of key results, and a funding application for a full trial may be undertaken if criteria for progression are met.

## 3 DISCUSSION

The HH4P feasibility study will be the first to evaluate the practicality and utility of home‐based HIIT for PwP. Given the evidenced physiological and clinical benefits of HIIT in PwP^4^ and various populations,^5^, ^6^, ^7^ the potentially ameliorative mechanistic properties,^4^, ^5^, ^24^ and the barriers to exercise faced by PwP,^9^, ^10^ it would appear that HIIT, particularly in the home setting could be apposite for PwP. Moreover, evidence from a recent systematic review^19^ regarding the apparent feasibility of home‐based exercise of lower intensity for PwP appears to be encouraging.

Results from HH4P will inform the potential development and implementation of a definitive, fully powered home‐based HIIT trial for PwP with long‐term follow‐up, which could in turn provide further important evidence regarding practicality and effectiveness for this population, adding to the existing knowledge regarding exercise and Parkinson's.

Ultimately, if home‐based HIIT is evidenced to be effective and practical for PwP, this could have crucial implications for clinical practice and provide PwP with an effective and feasible exercise alternative to integrate into disease management strategy. This could result in reduced symptoms and improved health and well‐being for PwP, increased life expectancy, and reduced caregiver and economic burden. Conversely, if home‐based HIIT is found to be impractical for this population, it will inform a more streamlined direction for future research examining strategies for the implementation of high‐intensity exercise for PwP.

## AUTHOR CONTRIBUTIONS

All authors have read and approved the final version of the manuscript. Conrad Harpham had full access to all of the data in this study and takes complete responsibility for the integrity of the data and the accuracy of the data analysis.

## CONFLICT OF INTEREST STATEMENT

The authors declare no conflict of interest.

## TRANSPARENCY STATEMENT

The lead author Conrad Harpham affirms that this manuscript is an honest, accurate, and transparent account of the study being reported; that no important aspects of the study have been omitted; and that any discrepancies from the study as planned (and, if relevant, registered) have been explained.

## Supporting information

Supporting information.

## Data Availability

Data sharing is not applicable to this article as no new data were created in this study.
